# A new hybrid model for improving outlier detection using combined autoencoder and variational autoencoder

**DOI:** 10.1038/s41598-025-28976-6

**Published:** 2025-12-08

**Authors:** Ahmed M. Daoud, Osama M. Elkomy, Walid I. Khedr, Khalid M. Hosny

**Affiliations:** Department of Information Technology, Faculty of Computers and Informatics, Zagazig University, Zagazig, Egypt

**Keywords:** Anomaly detection, Autoencoder, Variational autoencoder, Hybrid model, Outlier detection, Computational biology and bioinformatics, Engineering, Mathematics and computing

## Abstract

In this paper, we propose a new hybrid model, called AVE, that integrates the strengths of Autoencoder (AE) and Variational Autoencoder (VAE) to enhance outlier detection for numerous high-dimensional datasets. The proposed architecture leverages the reconstruction strengths of AE and the regularized latent space of VAE to build a stable framework for anomaly detection. Our experimental evaluation is conducted on 16 standard test sets from various domains. The results show that the AVE architecture outperforms the standalone architecture of AE, VAE, and other compared algorithms. The AVE architecture, specifically, achieves an average precision of 0.6925 and an average ROC-AUC of 0.8902 across the subsets of this test set. It surpasses the second-best architecture by 25.99% for precision and 5.47% for ROC-AUC. It surpasses AE and VAE architectures by 67.26% and 45.44% in precision, and by 7.71% and 10.03% in ROC-AUC, respectively. It achieves the best accuracy on 12 of the 16 datasets and the optimal ROC-AUC on 5 of them. Our findings demonstrate that AVE is a more reliable and precise method for detecting outliers, particularly in complex, high-dimensional datasets, making it an effective solution for real-world applications.

## Introduction

Anomaly detection^1^ identifies anomalous behavior that differs significantly from normal behavior in a data stream. One of the significant challenges in anomaly detection is identifying actual outliers while minimizing detection errors^2^. Anomalies can be complex—they are often context-specific, rare, or highly complex—making them difficult to detect, especially in complex datasets or where normal and abnormal data don’t match. Moreover, the challenge becomes significantly more complicated when dealing with high-dimensional data. Many standard algorithms struggle to scale properly or confuse noise with anomalies, leading to erroneous conclusions. Furthermore, many real-world datasets lack labeled anomalies, so we often rely on unsupervised or semi-supervised learning methods.

Although various techniques are used to identify anomalies, there is a lack of methods for effectively distinguishing normal from anomalous data. This is due to the diversity of anomalies and the complexity of high-dimensional datasets. Anomaly detection faces numerous substantial challenges, such as:

- **Diversity of Anomalies:** Anomalies can take many forms, such as global, contextual, or collective outliers, requiring flexible detection systems.
- **High Dimensionality:** The "curse of dimensionality"^3^ can complicate the identification of pertinent information, as noise and irrelevant attributes may obscure the patterns we seek.
- **Class Imbalance:** The number of standard samples outweighs the number of outliers, as the outliers are rare in nature; this could affect model training and evaluation.
- **Lack of Labeled Data:** The absence of ground truth annotations for anomalies limits the application of supervised techniques.
- **Evaluation Biases:** Some standard evaluation metrics can introduce biases, making it difficult to compare different methods fairly.

Typically, people employ various traditional methods to detect anomalies, such as distance-based techniques (e.g., k-Nearest Neighbors, kNN), density-based methods (e.g., DBSCAN and Local Outlier Factor, LOF), and several other statistical approaches. Ensemble techniques and semi-supervised algorithms have recently helped enhance robustness, particularly when labeled data is limited. However, deep learning methodologies, especially AEs and VAEs, have shown strong potential in this domain, as they learn latent representations that capture complex structures. While these advancements are pretty impressive, there are still a few research gaps that we need to focus on:

1. Existing methods often struggle to generalize to unseen anomalies, particularly when datasets are diverse or noisy^4–6^.
2. There is relatively little research exploring how the strengths of different detection models can be combined to deliver consistently strong results across domains^7^.
3. The lack of well-defined benchmark datasets with standardized evaluation protocols limits fair comparison and reproducibility^8,9^.
4. Many deep learning approaches are demanding, creating scalability concerns in practical deployments.

Autoencoder (AE)^10,11^ and Variational Autoencoder (VAE)^10,11^ offer unique strengths for anomaly detection. AEs can effectively represent high-dimensional, nonlinear structures in data without requiring labeled samples. Then, it reconstructs the input data and uses the error between the input and reconstruction (e.g., reconstruction loss) to signal an anomaly. Although it is effective in many cases, it can be sensitive to noisy or overlapping data distributions. In contrast, VAE introduces a probabilistic mechanism for latent space regularization to overcome this limitation. It guarantees more organized and significant representations and improves the model’s generalization.

In this paper, we combine the capabilities of both AE and VAE to construct a new hybrid, powerful model called AVE. The AVE model is designed to efficiently handle the complexity of diverse, high-dimensional datasets. AVE improves detection performance without imposing excessive computational requirements. Tests on 16 publicly available datasets from diverse fields demonstrated that AVE outperforms independent AE and VAE models, as well as several leading comparison algorithms, in terms of accuracy and ROC-AUC. Based on our results, the strong generalization across diverse scenarios suggests that combining AE and VAE components is a promising approach for building robust, scalable anomaly detection systems.

The rest of this paper is organized into five sections: Section "Related work" presents an overview of related literature and methods. Section "Proposed methodology" goes into detail about the proposed AVE model. Section "Experimental setup and evaluation metrics" covers the experimental design, including datasets, competing approaches, and evaluation metrics. Section "Experimental results and discussion " summarizes the findings and discussion. Finally, section "Conclusion and future work" wraps up the paper with suggestions for future work.

## Related work

Advanced algorithms go beyond merely classifying observations as outliers or inliers by assigning scores that reflect the degree or probability of an observation being an outlier. Many models rely on various principles, such as the distance between objects^12^. Also, the density of the area around an object^13,14^, or the difference in the angles between object vectors^13^. Additionally, some methods apply alternative outlier detection principles to different domains^15^.

On the other hand, the ensemble techniques for improving classification are supported by well-established theory^16^. In unsupervised clustering, applying ensemble methods has a substantial history of empirical studies^17^. Moreover, combining different clustering outcomes plays a key role in ensemble clustering as a distinct technique and in related methods such as multi-view, subspace, and alternative clustering approaches. The ensemble approach has also been applied to combine clustering evaluation metrics. The recent semi-supervised anomaly detection methods^18^ have demonstrated significant improvements over unsupervised approaches by utilizing limited labeled anomalies and large amounts of unlabeled normal data. However, these approaches often fail to generalize to unseen anomalies, as they focus solely on fitting the labeled examples.

Proximity-based methods for OD don’t require training on the data. Some methods measure the rarity of the point, such as the distance to the k nearest neighbors (kNN)^19^. Different approaches calculate the density-based ratio with the LOF, which is the anomaly score for each data point^20^. It determines how a data point’s density differs locally from that of its surrounding neighbors. Locality is defined as the distance between the kNNs, which is then used to determine the local density. The anomalous data points are revealed by calculating their local density, which is lower than that of the surrounding data points. Kriegel et al.^13^ introduced an angle-based measure to detect outliers in high-dimensional data. This technique assigns an outlier score to each point by measuring the angular variation between that point and pairs of other points. When there is variance in angles between a point and other points, it is considered an outlier. This approach remains effective in high dimensions because angles are more stable as dimensionality increases^21^. However, both the basic and approximate versions of this method are computationally expensive, requiring 3 × and 4 × the time, respectively.

In contrast, Shyu et al.^22^ provide an effective means, Principal Component Analysis (PCA), of detecting outliers through linear dimensionality reduction via Singular Value Decomposition (SVD). This technique decomposes the covariance matrix into orthogonal vectors, known as eigenvectors, each associated with an eigenvalue. Eigenvectors associated with higher eigenvalues capture most of the data’s variance, enabling the construction of a low-dimensional hyperplane that effectively represents it. Outliers, however, stand out clearly from normal data points, becoming more evident on the hyperplane defined by eigenvectors with lower eigenvalues.

For Gaussian-distributed datasets, the Minimum Covariance Determinant (MCD)^23,24^ can detect outliers using a robust covariance estimator. Although MCD is primarily designed for Gaussian data, it can also be applied to unimodal, symmetric distributions; however, it is unsuitable for multimodal data, where fitting may fail. In such cases, projection pursuit methods are recommended. The detection process begins by fitting a minimum covariance determinant model and calculating the Mahalanobis distance to ascertain the degree of outliers in the dataset. Also, a One-Class SVM (OCSVM)^25,26^ is a machine learning approach that detects anomalies, especially when trained only on normal data. It creates a hyperplane in a high-dimensional space to contain the normal data points. This hyperplane ideally separates the normal data from the rest of the feature space, including anomalies.

As a meta-learning approach, the feature bagging detector^27^ trains multiple base detectors on different dataset subsamples. It enhances prediction accuracy and mitigates overfitting by averaging or combining methods. The features are sampled randomly from the complete set of available attributes, while the subsample size matches the source dataset. While LOF is the default base estimator, alternative estimators such as kNN and ABOD can also be employed. Feature bagging promotes diversity among base estimators by constructing subsamples by randomly selecting feature subsets.

The anomaly score for the ABOD^13,28^ is calculated by taking the variance of an observation’s weighted cosine values with respect to its neighbors. It supports two versions: Fast ABOD, which uses kNN for approximation, and Original ABOD, which requires processing of all points and has a time complexity of $O({n}^{3})$. Another effective algorithm, Isolation Forest (IF)^29,30^, operates by “isolating” observations. It depends on the random selection of features and the split values, which are drawn from their maximum and minimum values. The iterative splitting is structured as a tree, where the number of splits required to isolate a sample is proportional to the path length from the root to the terminating node. The average traversal depth across a forest of randomized trees indicates normal behavior and influences the decision function.

He et al.^31^ compute the outlier scores using a Cluster-Based Outlier Factor (CBLOF) approach. It processes a dataset alongside a cluster model generated by a clustering algorithm, categorizing clusters into small and large based on parameters alpha and beta. While the original approach suggested weighting the outlier factor by cluster sizes, this feature is turned off by default to prevent unexpected behavior, leading to the anomaly score being calculated based on the size of the associated cluster and the distance from the closest larger cluster center. Goldstein and Denge^32^ introduce an efficient unsupervised outlier-detection method, Histogram-Based Outlier Detection (HBOD). It assumes that features are independent and determines the outlierness using histograms. It has two modes: one for a predefined number of bins across all features, and another for automatic binning. Each feature uses an optimal number determined using the Brige-Rozenblat method.

In addition, Liu et al.^33^ present a generative approach for unsupervised outlier detection. It significantly improves upon traditional methods for high-dimensional, complex datasets. It generates outliers that enhance the classification boundary between normal and anomalous data. Its ROC-AUC values on Pima, Annthyroid, WDBC, SpamBase, Arrhythmia, and p53Mutant were 0.758, 0.699, 0.964, 0.627, and 0.751, respectively. Also, it performed impressively on several other datasets: PenDigits (0.976), APS (0.966), PageBlocks (0.903), Ionosphere (0.874), Waveform (0.836), HAR (0.972), Shuttle (0.907), and Stamps (0.908). However, computational efficiency is degraded with smaller datasets.

Moreover, Nasrullah et al.^34^ present a framework, LSCP, to improve the effectiveness of unsupervised detection. It combines a base detector selected based on local data relationships. It addresses the poor accuracy and stability problem caused by the inclusion of incompetent detectors. These problems are addressed by constructing a local region for every test instance and selecting the top-performing detector for that region. It has a precision score of 0.3952 and an average ROC score of 0.7761. These experiments were performed on 20 datasets and compared with seven baseline OD methods.

Furthermore, Zhao et al.^35^ proposed a new framework for outlier detection called SUOD, which is designed for large-scale, unsupervised use. Its primary objective is to provide efficient, scalable outlier detection at scale with minimal performance loss. The experiments were performed on real-world benchmark datasets, including MNIST and HTTP. The results show the SUOD’s capability to accelerate outlier detection. However, the pseudo-supervised approximation has limitations for specific models, resulting in a decrease in ROC performance from 0.83 to 0.71 on Annthyroid. Lastly, Liu et al.^36^ introduce an unsupervised anomaly detection framework called RCA. It aims to solve the problem of overfitting anomalies in DL models. It employs a collaborative training approach in which multiple AEs exchange subsets of data samples with lower reconstruction errors. It was tested on benchmark datasets. It achieves ROC-AUC scores of 0.917 for Vowels, 0.950 for Sensor, and 0.806 for Arrhythmia.

Many recent works in unsupervised OD try to address the challenge of high-dimensional or complex data. They primarily focus on learning non-linear embeddings or combining local geometry and global structure—for example, Liu et al.^37^ introduce the Local Projection Score (LPS), which, for each point, collects its k-nearest neighbors and projects the small neighborhood into a low-rank subspace. Then, it uses the nuclear norm of the projected neighborhood as an anomaly score. LPS is simple, interpretable, and robust for moderate neighborhood sizes. A complementary work is done by Liu et al.^38^, who propose an anomaly detector that is based on representative neighbors via a sparse self-representation matrix and spectral graph analysis. The learned matrix is converted into a similarity graph, and a spectral analysis is used to rank anomalies. This method removes the need to fix k and explicitly addresses high dimensionality through sparse variable selection. It produces interpretable scores and performs competitively on standard benchmarks. These methods explicitly compute linear subspace divergence and neighborhood structure to detect anomalies.

A more recent approach, Liu et al.^39^, uses a kernel preserving embedding method for OD. As the typical embedding using kernel PCA may fail to retain the relevant structure, Liu et al. introduce this method to optimize embedding using a kernel similarity matrix that captures non-linear relations among data points. After embedding, scoring is performed based on deviations in the embedded space. The kernel matrix size scales as $O({n}^{2})$ so that embedding optimization may require substantial memory. Another complementary work, Liu et al.^40^, proposes an OD method that combines local density estimation with global graph structure. It learns a density estimate for each point and embeds the data into a worldwide structural representation to capture the larger-scale geometry. This approach is suitable for datasets with a complex cluster structure, where anomalies are hidden in low-density regions but lie outside the global manifold.

Another recent effort has been to develop clustering-based, parameter-free outlier-detection algorithms to reduce sensitivity to hyperparameters such as k and top-n, which are common in distance- and density-based algorithms. The Local Coulomb Outlier Factor (LCOF)^41^ proposes a Coulomb’s law-inspired formulation to model forces between data points, utilizing natural neighbors to adaptively select the neighborhood parameter and employing the interquartile range (IQR) method for automatic thresholding. LCOF achieves completely parameter-free detection of global and local outliers and outlier clusters.

Recently, there has been interest in using DNNs in AE-based outlier detection approaches^42^. VAEs are used for unsupervised outlier detection. AEs encode the input data and reconstruct it using a decoder. They minimize the reconstruction error, which is usually higher for anomalous data points. VAE extends that approach by constructing a probabilistic framework that regularizes the latent representation. They map input data to distributions rather than to fixed representations, thereby better handling noisy or contaminated data. Our proposed hybrid model (AVE) differs and complements these approaches in several ways. First, instead of neighborhood graphs or kernel matrices, it learns non-linear relations using dual encoders (from AE and VAE) and a shared decoder. It scores anomalies based on reconstruction error in the latent space. Second, the VAE branch imposes probabilistic regularization on the latent space (via KL divergence). This leads to smooth, structured latents that complement the AE branch’s deterministic reconstruction. This fusion enables the capture of both local reconstruction patterns and global latent structure, which makes AVE well-suited for complex, high-dimensional data. Third, AVE avoids parameter sensitivity by replacing them with parameter-optimized network training and threshold selection procedures. Finally, AVE demonstrates robust generalization and scalability, as shown in the results.

Table 1 summarizes various outlier detection methods. It shows their fundamental principles, advantages, and limitations. It encompasses a range of approaches, including proximity-based methods, ensemble techniques, and deep learning models such as AE, VAE, as well as other modern frameworks, including MO-GAAL^33^, LSCP, and SUOD. The table is a quick reference for selecting the most suitable approach for an anomaly detection task.

**Table 1:** Summary of outlier detection methods: key advantages and limitations.

Methodology	Advantages	Limitations
Proximity-Based Methods (e.g., kNN, LOF)	Simple, intuitive, and requiring no training, effective in low dimensions	Computationally expensive in high dimensions; sensitive to parameter choice (e.g., k)
ABOD	Robust to high-dimensional data; stable variance of angles compared to distance measures	High computational complexity: $O({n}^{3})$ for original ABOD, $O({n}^{2})$ for approximations
PCA	Effective in detecting outliers via linear dimensionality reduction	Assumes linearity; less effective for multi-modal or non-linear data distributions
MCD	Robust covariance estimation for Gaussian/unimodal symmetric distributions	Limited to specific distributions; not suitable for multi-modal data
Feature Bagging (FB)	Enhances accuracy and mitigates overfitting by using diverse subsamples	Limited performance improvement with highly correlated features; computational overhead
HBOS	Efficient for high-dimensional data; assumes feature independence	The independence assumption limits applicability in correlated datasets and is sensitive to the binning strategy
IF	Computationally efficient; scales well to large datasets; interpretable via tree paths	Performance decreases for overlapping or complex anomalies
CBLOF	Incorporates clustering into outlier scoring; adaptable to varying cluster sizes	Sensitive to parameter tuning; cluster assumptions may not align with dataset characteristics
AEs	Captures data structure through reconstruction error, making it effective for high-dimensional anomaly detection	Struggles with noisy or incomplete data; prone to overfitting anomalies
VAEs	Regularizes the latent space; handles noisy, complex data distributions well	Requires careful tuning of the probabilistic framework; slightly more complex than AEs
MO-GAAL	Combines multiple sub-generators, avoids mode collapse, and is robust on complex datasets	Computationally intensive for small datasets; requires iterative sub-generator training
LSCP	Improves accuracy and stability by selecting locally competent base detectors	Depending on the local region definition, there is a higher computational cost due to dynamic detector selection
SUOD Framework	Scales unsupervised OD with minimal accuracy loss; efficient for large-scale tasks	Approximation techniques may reduce performance for specific models (e.g., ABOD)
RCA	Mitigates overfitting by focusing on cleaner subsets of data and effectively handling noise and missing values	Relatively new; requires further validation across diverse datasets and real-world scenarios

## Proposed methodology

We propose AVE, an approach that combines the strengths of the autoencoder (AE) and the variational autoencoder (VAE). This approach combines AE’s reconstruction capabilities with VAE’s probabilistic latent-space modeling, thereby increasing the robustness of anomaly detection.

### Autoencoder

**Fig. 1:**
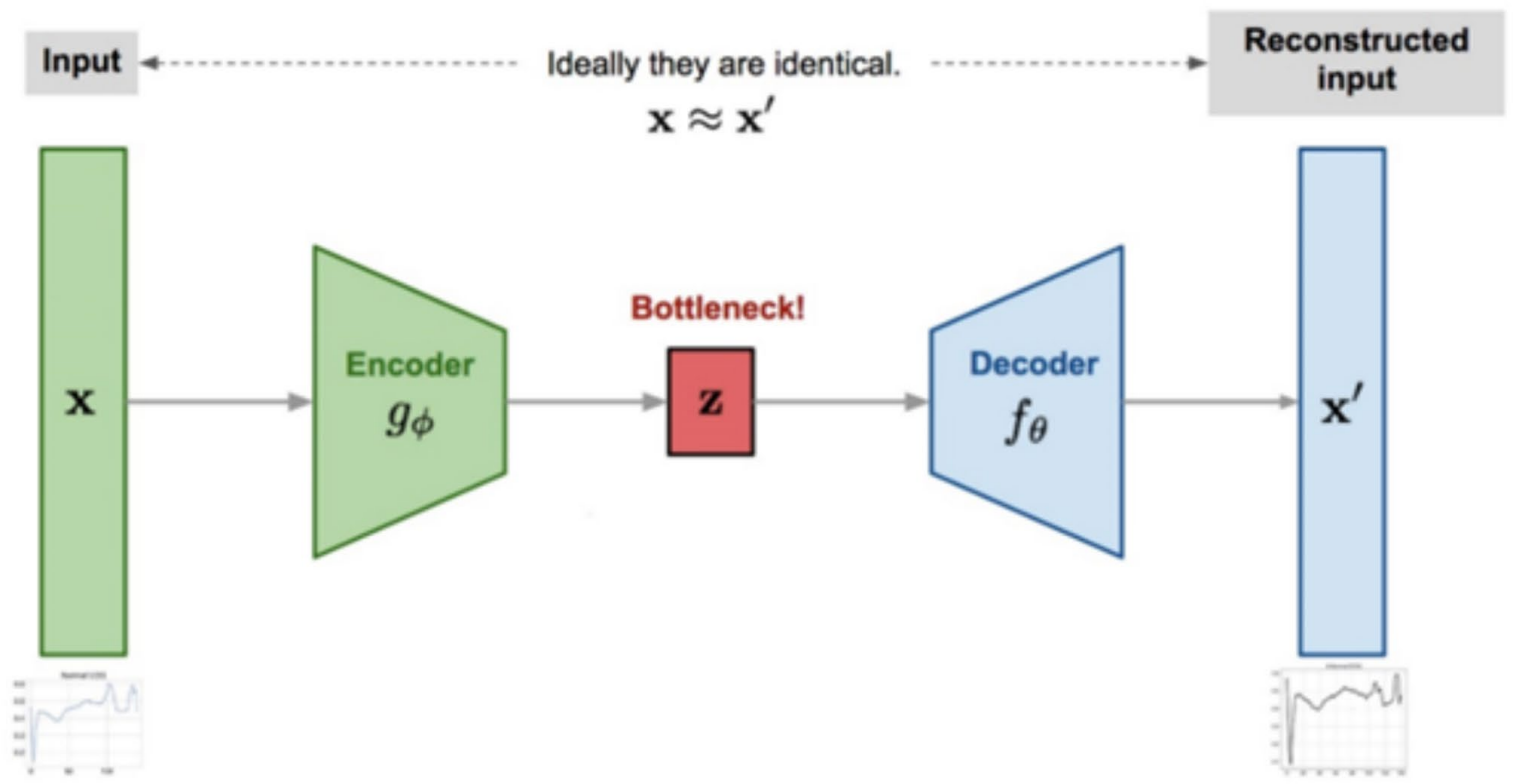
Autoencoder architecture (encoder-latent space-decoder).

An autoencoder (AE) is a neural network designed for unsupervised anomaly detection tasks, comprising two primary components: an encoder and a decoder. The decoder then reconstructs the data, attempting to match the original input as closely as possible while minimizing loss. Anomaly detection is based on reconstruction error, the difference between the input and the reconstructed data. Data points that have high reconstruction errors are marked as outliers. The primary objective of an autoencoder is to minimize the discrepancy between the original input and its reconstructed output, typically measured using a loss function such as mean squared error. That function penalizes the differences between the input denoted by $X$ and the output version denoted by $X{\prime}$. The optimization method enables the model to learn the essential features in the data while maintaining reconstruction accuracy. Figure 1 illustrates the main structure of an AE.

When the AE has one hidden layer with a linear activation function, it performs a linear transformation like PCA^43^, where the components correspond to the neurons in that layer. For deeper architecture, multiple hidden layers and non-linear activation functions are needed to detect complex and non-linear relationships. Hence, several important hyperparameters, such as the number of hidden layers, the number of neurons per layer, the activation functions, and the regularization techniques, must be fine-tuned to improve anomaly detection accuracy. The autoencoder’s ability to learn without supervision makes it a valuable tool for detecting outliers in various applications.

The recent advancements have positioned autoencoders as a leading approach in anomaly detection research. Unlike traditional methods, autoencoders can learn hidden representations without labeled data, making them suitable for handling unsupervised tasks. They can capture hidden patterns and model complex relationships, enabling them to detect subtle anomalies in high-dimensional datasets effectively. Autoencoders are valuable tools, but they have some limitations. Their success depends on the selection of their architecture and hyperparameters. Additionally, they require extensive and varied training datasets to function optimally. Inadequate data and inappropriate tuning could compromise their ability to detect anomalies. Their flexibility and ability to reveal hidden patterns make them essential in today’s anomaly detection methods.

Autoencoders operate according to specific equations for the encoding, decoding, and reconstruction steps. These equations demonstrate how the input data evolves as it passes through the network and illustrate how the model fine-tunes its parameters.

- Encoding Function

Equation (1)^44^. shows how the encoder takes the input $\text{X}$ and converts it into a compact representation $\text{Z}$ in a lower-dimensional latent space:

1 $$Z={f}_{enc}\left(X\right)= \sigma ({W}_{enc} X+ {b}_{enc})$$

${W}_{enc}$ Is the weight matrix of the encoder, ${b}_{enc}$ represents the encoder’s bias vector, and $\sigma$ is the activation function (such as ReLU or the sigmoid function).

- Decoding function

Equation (2)^44,45^. shows how the decoder takes the latent space $\text{Z}$ and uses it to reconstruct the output:

2 $${X}{\prime}={f}_{dec}\left(Z\right)= \sigma ({W}_{dec} Z+ {b}_{dec})$$

${W}_{dec}$ Is the weight matrix of the decoder, ${b}_{dec}$ represents the decoder’s bias vector, and $\sigma$ is the activation function (e.g., ReLU, sigmoid)*.*

- Reconstruction error (Loss function)

The reconstruction error quantifies how well the autoencoder reproduces the input:

Equation (3) shows the calculation of **MSE**:

3 $${\ell}(X,{X}{\prime})= \frac{1}{N} {\sum }_{i=1}^{N}{\left({X}_{i}- {{X}_{i}}{\prime}\right)}^{2}$$

Other loss functions, such as Binary Cross-Entropy (BCE) for binary data, can also be used as shown in Equation (4):

4 $${\ell}(X,{X}{\prime})= \frac{1}{N} {\sum }_{i=1}^{N}\left({X}_{i}\text{log}\left( {{X}_{i}}{\prime}\right)+(1- {X}_{i})log(1- {{X}_{i}}{\prime}\right))$$

where $X$ is the original input data, ${X}{\prime}$ the reconstructed data from the model, ${X}_{i}$ the ith input sample, ${{X}_{i}}{\prime}$, the ith reconstructed sample corresponding to ${X}_{i}$, $N,$ the Total number of samples.

- Optimization

Equation (5) shows how the autoencoder optimizes the weights and biases ${W}_{enc}$, ${W}_{dec}$, ${b}_{enc}$, ${b}_{dec}$:

5 $${min}_{w,b} {\ell}(X,{X}{\prime})$$

Here ${min}_{w,b}$ is the objective of minimizing the loss concerning weights and biases, ${\ell}(X,{X}{\prime})$ is the Loss function.

- Gradient descent update

During training, the weights and biases are updated using gradient descent as shown in Eq. (6):

6 $$W \leftarrow W - \eta \frac{\partial \ell }{{\partial W}}b \leftarrow b - \eta \frac{\partial \ell }{{\partial b}}$$

$W$ is the weight matrix of the model, $\eta$ is the learning rate (eta), $\frac{\partial {\ell}}{\partial W}$ is the gradient of ${\ell}$ w.r.t. weights, $\frac{\partial {\ell}}{\partial b}$ The gradient of ${\ell}$ w.r.t. biases, the arrow ⟵ indicates updating the value.

### Variational autoencoder

VAEs are a type of neural network that does more than reconstruct data. It learns a probabilistic representation of the data. Unlike autoencoders, which compress inputs into fixed latent vectors, VAEs map them to a Gaussian distribution. The encoder produces the mean and variance that characterize this latent space. To generalize effectively to unseen data samples, the decoders reconstruct the data using samples from the learned distribution. The main strength of VAE lies in its capability to conduct a dual-objective optimization process. First, it minimizes reconstruction loss to ensure the output is as close as possible to the input. Second, it minimizes the Kullback–Leibler (KL) divergence, which forces the learned distribution to align with a predefined standard distribution. This process creates a meaningful latent space that can be used to reconstruct new data points similar to those in the original dataset.

Thanks to their ability to model complex distributions and generate realistic outputs, VAEs have become essential tools for a range of generative tasks, including image synthesis, data augmentation, and anomaly detection. Their structured latent space makes them suitable for analyzing high-dimensional data, discovering patterns, and exploring relationships within datasets. However, to achieve optimal performance, you must determine the right balance between reconstruction loss and KL divergence during training and ensure that the architecture is specifically designed for the task.

In VAEs, a probabilistic approach is used, where the reconstruction probability is shown in Eq. (7)^46^.:

7 $$\ell_{VAE} = {\mathbb{E}}_{q(Z|X)} \left[ { - \log p(X|Z)} \right] + KL(q\left( {Z|X} \right)||p(z))$$

where ${\mathbb{E}}_{q(Z|X)}\left[-\text{log}p(X|Z)\right]$ is the reconstruction loss (used to penalize poor reconstructions), $KL (q\left(Z|X\right) || p(z))$ calculates the KL divergence,$\text{q}(Z|X)$ is the learned distribution, $p(z)$ is the prior distribution of the latent space ∼$N(\text{0,1})$.

### Advantages of the hybrid model

- Autoencoder (AE):
  - It compresses the input data into a simpler compressed form and then reconstructs it.
  - Good at learning patterns in normal data and identifying large deviations from this pattern in the data that might point to anomalies.
- Variational Autoencoder (VAE):
  - Learns a probabilistic representation of the latent space.
  - Modeling the mean and variance can handle uncertainty in the data, making it more robust against variations in normal data.
  - Helps find complex variations in the data that AE might miss.
- Hybrid Model:
  - Combines both the deterministic pros of (AE) and the probabilistic (VAE) latent representations.
  - The VAE helps model the uncertainty of normal data and the underlying distribution, while the AE gives higher reconstruction accuracy.
  - Gives better generalization and detection accuracy, which is useful in distinguishing between normal data and anomalies.
  - The model uses KL divergence loss for regularization, which helps avoid overfitting and captures the underlying data distribution.

### Proposed model architecture

**Fig. 2:**
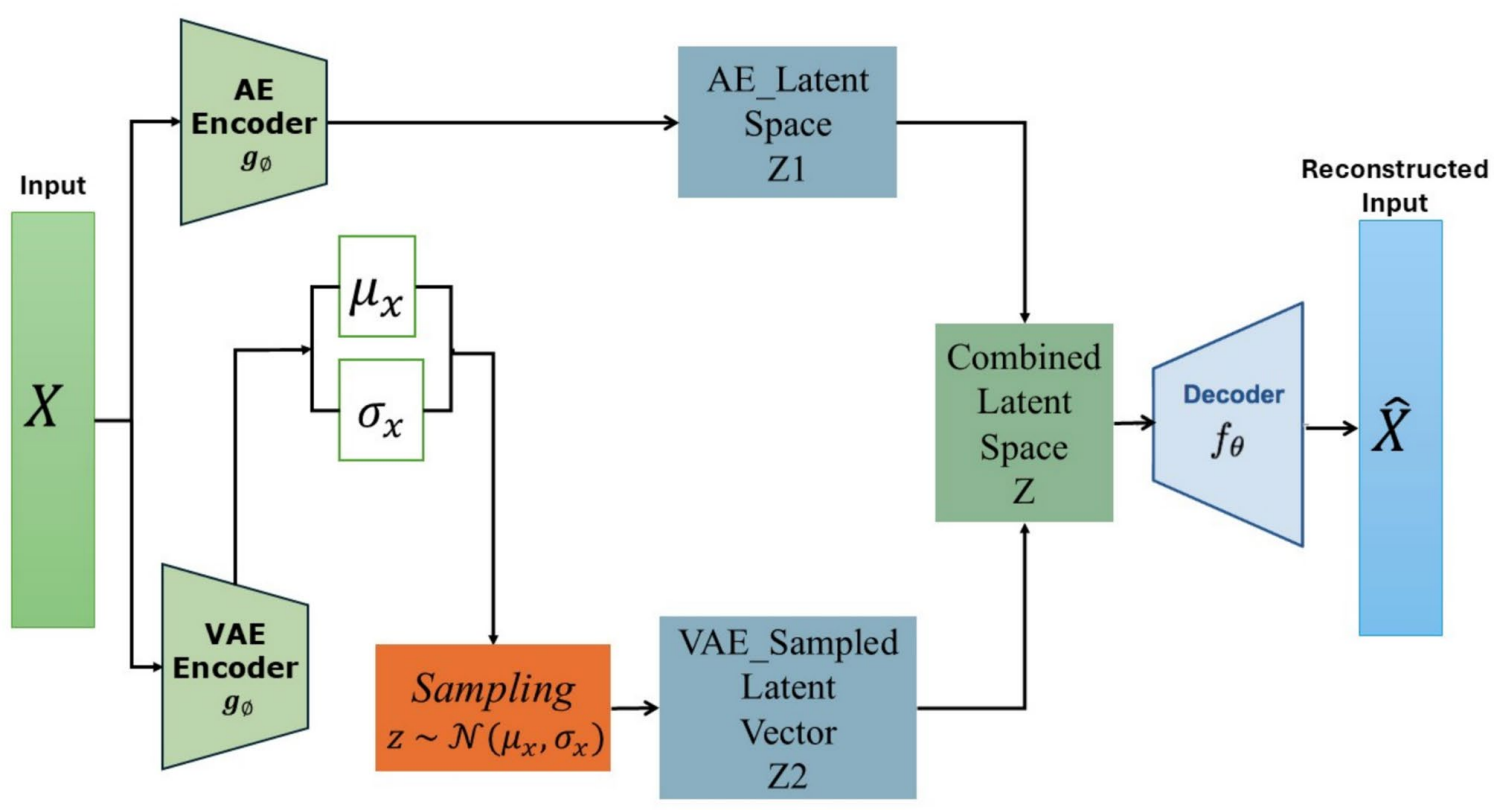
The AVE architecture diagram.

The architecture diagram for AVE is shown in Fig. 2, combining the strengths of AE and VAE. It has two separate encoder branches for the AE and VAE. A combined decoder follows it to reconstruct the original data. The VAE branch introduces a sampling layer to a stochastic latent space, ensuring that the latent variables are regularized to follow a normal distribution. In contrast, the AE branch captures the data’s deterministic features. The sampling layer modifies the latent variables through a differentiable transformation, enabling backpropagation through the stochastic latent space. The sampling operation is given by Eq. (8):

8 $${z}_{2}= {\mu }_{x}+ {e}^{{\sigma }_{x}}\times \epsilon$$

where ${z}_{2}$ is the reparameterized latent vector used for decoding, ${\mu }_{x}$ Is the mean of the latent variable distribution, ${\sigma }_{x}$ The standard deviation of the latent distribution, $\epsilon \sim N (\text{0,1})$, represents an arbitrary variable drawn at random from a normal distribution. Both AE’s latent space and the VAE’s sampled latent vector are combined into an averaged combined latent space $z$. The combined latent is fed to a single shared decoder. We also evaluate alternative fusion mechanisms (concatenation and weighted sum) in the sensitivity analysis to KL Weight (Sect. 5.2). Averaging gave the best trade-off between reconstruction capability and anomaly discrimination in our benchmarks.

During the training process, the model tries to minimize two loss functions: the $MS{E}_{loss}$ and the ${\text{KL}}_{\text{loss}}$. The $MS{E}_{loss}$ is calculated, using Eq. (9), as the MSE between the input, denoted as $x$, and the output, denoted as $\widehat{x}$. The ${\text{KL}}_{\text{loss}}$, shown in Eq. (10), is used to organize the VAE’s latent space. It is considered a regularizer that ensures the distribution learned by the encoder is not too far from the normal distribution (penalizing the difference).

9 $$MS{E}_{loss}= \frac{1}{N} {\sum }_{i=1}^{N}{\left({x}_{i}- {\widehat{x}}_{i}\right)}^{2}$$

10 $${\text{KL}}_{\text{loss}}= -0.5 \times mean ({\sigma }_{x}- {{\mu }_{x}}^{2}- {e}^{{\sigma }_{x}}+1)$$

The total loss is a weighted sum of the $MS{E}_{loss}$ and the ${\text{KL}}_{\text{loss}}$. This balance controls the model’s capability to reconstruct better data and to keep the latent space properly regularized, as shown in Eq. (11):

11 $${\text{Total}}_{\text{loss}}= MS{E}_{loss}+0.1 \times {\text{KL}}_{\text{loss}}$$

This deterministic AE and stochastic VAE encoding combination provides a robust model for anomaly detection and data reconstruction.

For thresholding, we use a dataset-adaptive contamination estimate derived from the dataset labels. We compute the outlier fraction as the number of anomalies divided by the total number of samples ($N$) in Eq. 12 and set the contamination rate by dividing the resulting outlier fraction by 3 in Eq. 13. This conservative choice reduces the chance of overestimating anomalies during thresholding.

12 $$Outlier fraction= \frac{Number of anomalies }{N}$$

13 $$Contamination rate (c)= \frac{Outlier fraction}{3}$$

The proposed AVE’s novelty lies in:

1. End-to-end joint training of dual encoders with a shared decoder.
2. Averaged deterministic and stochastic latent representation.
3. KL-weighted regularization balancing the two latent spaces.
4. Integrated adaptive thresholding and feature selection pipeline.

### Flow chart

**Fig. 3:**
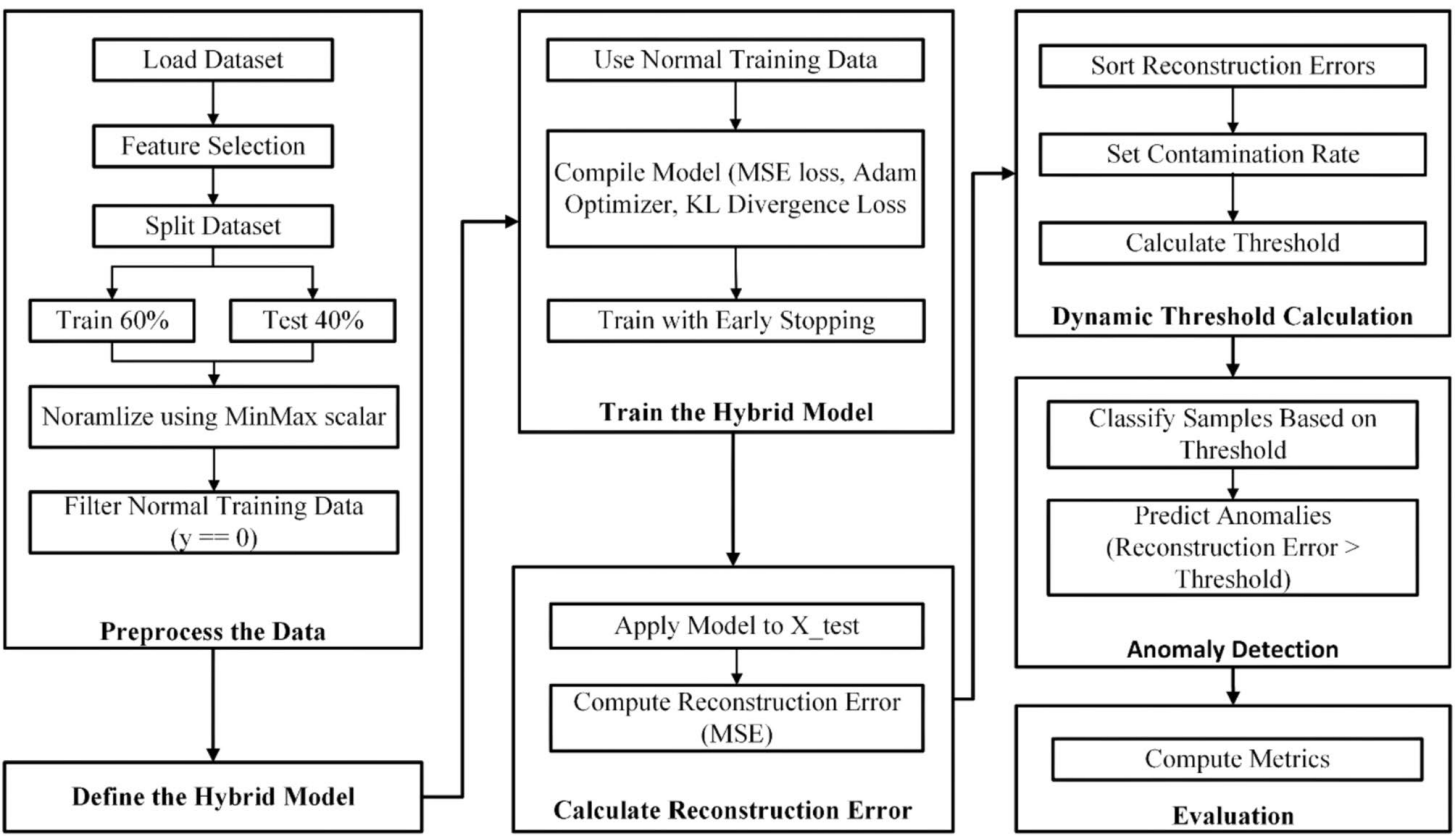
Detailed flow chart of the proposed framework.

The detailed flowchart of the entire framework is shown in Fig. 3, which starts with data preprocessing, where the dataset is loaded. Next, it performs feature selection to extract the most relevant features. The dataset is divided into two distinct subsets: a training set (60%) and a test set (40%). Then, we apply the MinMax normalization technique to rescale the extracted features, ensuring they lie within a fixed range. Finally, the normal data (samples with y = 0) are filtered for training. Next, define the hybrid model, consisting of an AE and a VAE, each with its own encoder. The VAE branch includes a reparameterization layer, and both branches share a decoder. In the training phase, we train the model on the normal data using MSE loss, the Adam optimizer, and a KL divergence loss with a small weight. Once trained, the model calculates the reconstruction error on the test data by comparing the original and reconstructed values. Next, we determine a dynamic threshold by sorting the reconstruction errors in ascending order. Based on the assumed contamination rate (the percentage of anomalies), the threshold is set to the point at which anomalies first appear in the sorted list. It adapts to the distribution of reconstruction errors, making it adaptable across different datasets. Samples with reconstruction errors exceeding the selected threshold are classified as anomalies during the anomaly detection stage. Finally, we evaluate the model’s accuracy, precision, recall, F1 Score, and ROC-AUC.

### Pseudo code

Algorithm: Hybrid Autoencoder-VAE for Anomaly Detection
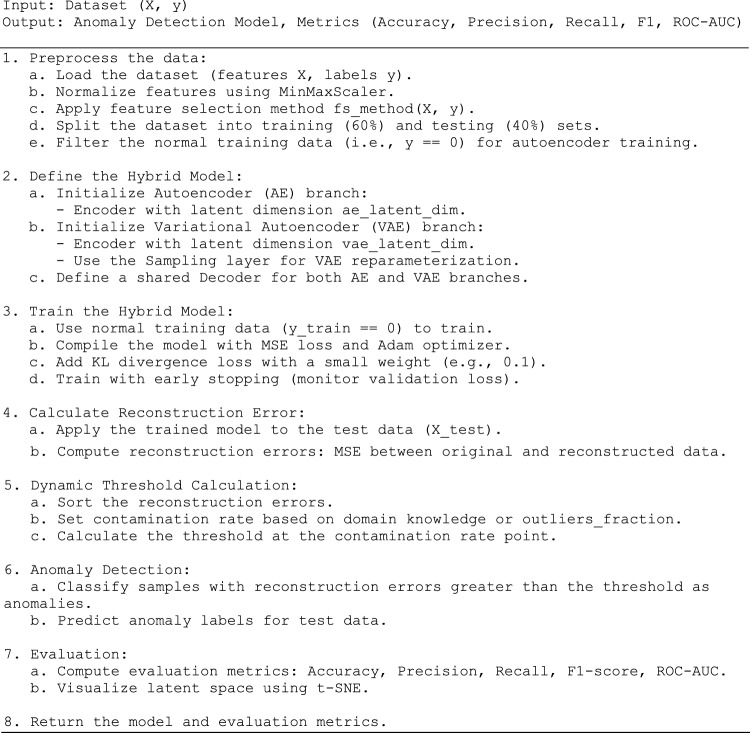


### Code availability

The custom code developed for this paper is shared openly in the GitHub repository https://github.com/A7medDaoud/AVE-Hybrid-Anomaly-Detection, in which all scripts and implementation details are provided to reproduce the results reported in this paper. A permanently archived version of this repository is available via Zenodo at (10.5281/zenodo.17583881). Both versions correspond to the release used in this publication and contain all the necessary scripts to reproduce the experiments. The code is distributed under the MIT License.

## Experimental setup and evaluation metrics

This section provides an overview of the details of a set of public benchmark datasets and describes the competing algorithms used. Next, discuss the parameter configurations used for experimenting with the AVE model and other approaches. The performance metrics are then provided to demonstrate the effectiveness of the proposed model. Finally, describe the device specifications used to perform all experiments.

### Dataset

The evaluation of unsupervised OD algorithms still has a lot of challenges in research^8^, particularly regarding the biases associated with the commonly used assessment. These biases complicate comparisons between new and established methods, hindering a clear understanding of their strengths and weaknesses. It is also difficult to evaluate different OD approaches since there are not enough benchmark datasets^8,9^ with annotated labels. A further challenge in this context is the absence of widely accepted benchmark datasets. Emmott et al.^47^ proposed an approach for creating benchmark datasets using the “classification hardness” concept as the basis. Their procedure was proper, but it didn’t provide details on their experiments, and the datasets weren’t available for use. Next, Campos et al.^8^ performed an experimental study to characterize the datasets that are going to be used for OD and discuss their effectiveness as a benchmark dataset. They also suggest adapting existing evaluation measures to enhance their applicability in assessing OD performance.

A typical method for comparing OD models is to evaluate their performance against classification datasets^8^. When semantic labeling is absent, the ground truth is established by selecting a small percentage of data from the minority classes and treating them as outliers. At the same time, the rest of the classes are treated as inliers^9^. This down-sampling approach for creating datasets has been previously adopted by researchers, including^12,27^, and^48^. This approach aligns with the “outlierness” concept, in which outliers are drawn from classes that differ from those of the inliers. Our research builds on these studies to emphasize the novelty and contribution by developing a hybrid deep neural network model that combines AE and VAE to enhance outlier detection accuracy and evaluating it against OD models.

Multidimensional (or tabular) data is common in real-world applications, so we concentrate on the publicly available high-dimensional datasets. We use 16 real-world datasets, as described in Table 2, to explicitly evaluate the performance of anomaly detection. These datasets span different domains and are labeled to indicate whether each instance is an outlier. Each dataset contains an outlier portion ranging from 1.22% to 35.9%. For real-world scenarios, we first split each dataset into two subsets: 60% for training and 40% for testing.

**Table 2:** Selected publicly accessible real-world high-dimensional benchmark datasets.

No	Dataset	Domain	Number of samples (n)	Dimension (d)	Number of outliers (%)	Description (outlier vs. normal)	Ref
1	Arrhythmia	Healthcare	452	274	66(14.6)	12 Types of cardiac arrhythmia vs. healthy	^48^
2	Cardio	Healthcare	1831	21	176(9.61)	Pathologic, suspect vs. healthy	^48^
3	Glass	Forensic	214	7	9(4.21)	Class 6 vs. others	^8^
4	Ionosphere	Oryctognosy	351	33	126(35.9)	Class vs. Class’ g’	^48^
5	Letter	Image	1600	32	100(6.25)	Outliers vs. normal	^49^
6	Lympho	Healthcare	148	18	6(4.05)	Classes 1 and 4 vs. others	^50^
7	Mnist	Image	7603	100	700(9.21)	digit-six classes vs. digit-zero class	^51^
8	Musk	Chemistry	3062	166	97(3.17)	Classes 213 and 211 vs. classes j146, j147, and 252	^52^
9	Optdigits	Image	5216	64	150(2.88)	digit 0 vs digits 1–9	^53^
10	Pendigits	Image	6870	16	156(2.27)	Class ‘4’ vs. others	^54^
11	Pima	Healthcare	768	8	268(34.9)	Diabetes vs. healthy	^48^
12	Satellite	Astronautics	6435	36	2036(31.64)	Classes’ 2, 4, 5’ vs. others	^55^
13	satimage-2	Astronautics	5803	36	71(1.22)	Class’ 2’ vs. others	^50^
14	Shuttle	Astronautics	49,097	9	3511(7.15)	Others vs. Class ‘1’	^56^
15	Vowels	Linguistics	1456	12	50(3.43)	Class’ 1’ vs. Classes’ 6, 7, and 8’	^8^
16	Wbc	Healthcare	278	30	21(5.56)	Malignant vs. Benign	^50^

### Competing methods

We use the benchmark models from^57^. The proposed model is compared to 18 baseline models from similar related areas, including Proximity-based models (KNN, LOF, CBLOF, and HBOS), Linear Models OCSVM, PCA, and MCD), Probabilistic Model (ABOD), Ensemble Models (FB, IF, and SUOD), and Neural Network Models (AE, VAE, LUNAR^58^, RCA, MO_GAAL, DAGMM, and Deep SVDD), as shown in Table 3.

**Table 3:** OD baseline models used for comparison.

Ref	Model	Category
^11^	VAE	Neural network
^19^	kNN	Proximity (distance-based)
^20^	LOF	Proximity (density-based)
^24^	MCD	Linear model
^25^	OCSVM	Linear model
^27^	FB	Ensemble
^28^	ABOD	Probabilistic
^30^	IF	Ensemble
^31^	CBLOF	Proximity
^32^	HBOS	Proximity
^35^	SUOD	Ensemble
^43^	PCA	Linear model
^36^	RCA	Neural network
^33^	MO_GAAL	Neural network
^59^	DAGMM	Neural network
^60^	Deep SVDD	Neural network
^44^	AE	Neural network
^58^	LUNAR	Neural network

The ReLu activation function $g(a) = max(0,a)$ is used. And ℓ2-norm regularization with λ = 0.001 is applied to avoid overfitting. The training parameters are provided in Table 4.

**Table 4:** Summary of training parameters used in model development.

Parameter	Values
Batch size	64
Optimizer	Adam
Epochs	100
Loss function	MSE
Learning rate(lr)	0.0001
Early stopping	True, patience = 5

### Performance evaluation metrics

Our experiments use two popular, recognized performance metrics: ROC-AUC and precision^9^ and^33^. ROC-AUC, which summarizes the true positives versus false positives across different thresholds, is a popular metric for its interpretability and ability to handle class imbalance. We calculate the average ROC-AUC using 10 independent trials, each with 60% of the data reserved for training and 40% for testing. ROC-AUC and precision are widely used metrics for evaluating OD models. These evaluation metrics are used due to their robustness and complementary insights into model performance.

- Confusion Matrix: In the context of OD, the outliers represent the positive class, while the normal data represent the negative class. Table 5 shows the confusion matrix for OD, which summarizes the counts of correctly and incorrectly classified outliers and normal instances.

**Table 5:** Confusion matrix for OD.

	Predicted outlier	Predicted normal
Actual outlier	Correctly identified (TP)	Missed detection (FN)
Actual normal	False alarm (FP)	Correctly rejected (TN)

- In OD, precision indicates the fraction of correctly detected outliers (true positives) to all instances classified as outliers.where TP: is the actual outliers correctly identified, and FP: is the normal instances incorrectly identified as outliers.
  14 $$Precision= \frac{TP}{TP + FP}$$

- The ROC-AUC is derived from the ROC curve, which plots the TPR against the FPR.where FN: is the misclassified outliers as usual, i.e., missed anomalies, and TN: is the normal instances correctly identified as usual.
  15 $$TPR= \frac{TP}{TP + FN}$$
  16 $$FPR= \frac{FP}{FP+ TN}$$

Then, the ROC-AUC is computed by plotting TPR vs. FPR at all possible classification thresholds. It reflects the probability that an outlier receives a higher score from the model than a randomly chosen standard instance.

In our evaluation, we compute precision as shown in Eq. (14) and derive the ROC-AUC by plotting the actual TPR (Eq. (15)) against the FPR (Eq. (16)) across all thresholds. These metrics are chosen for their sensitivity to outlier detection effectiveness, particularly in cases of class imbalance.

### Device specifications

We performed the experiments on a single laptop to ensure reproducibility and a fair performance evaluation. The system ran Windows 10 with an Intel(R) Core i7-4500U CPU at 1.80GHz and 2.40GHz, 8 GB of RAM, and a single-core setup to maintain consistent baseline performance. All coding and testing were performed in Visual Studio Code using Python 3.10.1.

## Experimental results and discussion

This section presents the results, compares the AVE model against baseline methods using ROC-AUC and precision metrics, and concludes with a discussion of the findings. Table 6 compares the performance of various OD algorithms (18) and the proposed AVE model across multiple datasets using the ROC-AUC metric, which measures the model’s ability to separate normal instances from anomalous ones. Table 7 analyzes performance using the precision metric, which measures the model’s ability to identify anomalous data points relative to the true predictions. Our analysis shows that AVE outperforms other models, including AE, VAE, and others. The table includes ROC-AUC scores for 16 datasets with varying characteristics, such as sample size, dimensionality, and outlier proportion.

**Table 6:** ROC-AUC comparison of AVE against 18 baseline algorithms across 16 real-world datasets.

Dataset No	ABOD	CBLOF	FB	HBOS	IForest	KNN	LOF	MCD	OCSVM	PCA	LUNAR	SUOD	AE	VAE	Deep SVDD	MO GAAL	RCA	DAG MM	(AVE)
1	0.7688	0.7715	0.7793	0.8219	0.8082	0.7861	0.7787	0.779	0.7812	0.7815	0.7978	0.8096	0.7678	0.773	0.8236	0.751	0.8361	0.8373	**0.845**
2	0.5692	0.7151	0.5904	0.8351	0.9249	0.7236	0.5736	0.8275	0.9348	0.9504	0.5544	0.9024	0.7807	0.9603	0.9626	N/A	0.9711	**0.9713**	0.9621
3	0.7951	0.8514	0.8605	0.7389	0.7286	0.8508	**0.8644**	0.7853	0.6324	0.6747	0.801	0.7477	0.8152	0.6825	0.671	N/A	0.7008	0.7513	0.7788
4	0.9248	0.9233	0.8721	0.5614	0.8441	0.9267	0.8753	**0.9554**	0.8419	0.7962	0.9322	0.8693	0.9314	0.8355	0.7914	0.874	0.898	0.8898	0.8962
5	0.8782	0.8427	0.8659	0.5927	0.6261	0.8766	0.8594	0.8089	0.6118	0.5283	**0.9076**	0.6764	0.8495	0.5895	0.5661	N/A	0.5393	0.6217	0.7282
6	0.911	0.9681	0.9766	0.9957	0.9881	0.9745	0.9771	0.8964	0.9759	0.9847	0.9434	0.9839	0.9787	0.9851	0.9802	N/A	0.9953	**1**	0.9944
7	0.7815	0.8298	0.7156	0.5742	0.7993	0.8481	0.7161	0.86	0.8529	0.8527	0.7444	0.832	0.8431	0.9021	0.8347	N/A	**0.9578**	0.9508	0.8835
8	0.1844	0.8763	0.5264	**1**	0.9984	0.7986	0.5287	**1**	**1**	**1**	0.6566	0.9591	0.9175	**1**	0.9997	N/A	**1**	**1**	**1**
9	0.4667	0.3524	0.447	0.8733	0.7077	0.3708	0.45	0.3895	0.4997	0.5086	0.4539	0.6697	0.4649	0.5005	0.6914	N/A	0.7959	0.8119	**0.9939**
10	0.6878	0.7624	0.4727	0.9238	0.947	0.7486	0.4698	0.8332	0.9303	0.9352	0.6671	0.8909	0.7953	0.9346	0.8901	**0.9760**	0.9202	0.9151	0.815
11	0.6794	0.6556	0.6186	0.7	0.6767	0.7078	0.6271	0.6747	0.6215	0.6481	0.6804	0.6655	0.6455	0.556	0.6204	**0.7580**	0.4177	0.528	0.704
12	0.5714	0.6732	0.5566	0.7581	0.7065	0.6836	0.5573	0.8003	0.6622	0.5988	0.6196	0.7074	0.6562	0.7399	0.609	N/A	0.7318	0.7304	**0.8007**
13	0.819	0.9893	0.4571	0.9804	0.9949	0.9536	0.4577	0.9959	0.9968	0.9822	0.851	0.9919	0.9469	0.986	0.9806	N/A	0.9855	0.9877	**0.9977**
14	0.6235	0.6834	0.4708	0.9855	**0.9971**	0.6537	0.5264	0.9903	0.9917	0.9898	0.6353	0.9955	0.9931	0.9925	0.9862	0.907	0.979	0.979	0.988
15	0.9606	0.957	0.9372	0.6727	0.7588	**0.968**	0.941	0.7917	0.7802	0.6027	0.9498	0.8021	0.9188	0.6244	0.5354	N/A	0.5625	0.6592	0.9069
16	0.9047	0.9321	0.9338	**0.9516**	0.9246	0.9366	0.9349	0.9159	0.9319	0.9159	0.9252	0.943	0.9194	0.8829	0.9174	N/A	0.8612	0.8704	0.9495
Avg	0.7204	0.7990	0.6925	0.8103	0.8394	0.8005	0.6961	0.8317	0.8154	0.7969	0.7575	0.8404	0.8265	0.8091	0.8037	N/A	0.8220	0.8440	**0.8902**
Max Count	0	0	0	2	1	1	1	2	1	1	1	0	0	1	0	2	2	3	**5**

**Table 7:** Precision comparison of AVE against 18 baseline algorithms across 16 real-world datasets.

Dataset No	ABOD	CBLOF	FB	HBOS	IForest	KNN	LOF	MCD	OCSVM	PCA	LUNAR	SUOD	AE	VAE	Deep SVDD	MO GAAL	RCA	DAG MM	(AVE)
1	0.3808	0.4427	0.4379	0.5111	0.4679	0.4464	0.4334	0.3995	0.4614	0.4613	0.4643	0.4995	0.3997	0.4395	0.5008	N/A	0.5226	0.5194	**0.7625**
2	0.2374	0.3406	0.1643	0.4476	0.511	0.3323	0.1541	0.4469	0.5011	0.609	0.2515	0.4063	0.3402	0.6909	0.7145	N/A	0.8015	0.7819	**0.9826**
3	0.1702	0.0726	0.181	0	0.0726	0.0726	0.1476	0	0.1726	0.0726	0.1726	0.0726	0.1952	0.0726	0.0726	N/A	0.1559	0.1559	**0.2**
4	0.8442	0.8396	0.7017	0.3295	0.6369	0.8602	0.7063	0.883	0.7	0.5729	0.8644	0.7016	0.8273	0.585	0.6163	N/A	0.7118	0.6978	**1**
5	0.3801	0.2718	0.3536	0.0715	0.0772	0.3312	0.3641	0.1979	0.151	0.0875	**0.4295**	0.1051	0.3152	0.1088	0.1001	N/A	0.1684	0.1858	0.3769
6	0.4483	0.7517	0.7517	0.8467	0.8017	0.7517	0.7517	0.3567	0.7517	0.7517	0.6517	0.7017	0.6683	0.7517	0.7267	N/A	0.8267	**1**	0.0425
7	0.3555	0.3884	0.3313	0.1188	0.2998	0.4204	0.3343	0.2899	0.3962	0.3846	0.3493	0.3686	0.3958	0.468	0.4778	N/A	0.6531	0.6089	**0.7432**
8	0.0507	0.5863	0.2071	0.9783	0.9212	0.2733	0.1696	0.9921	**1**	0.9799	0.1294	0.4049	0.3592	0.9916	0.9794	N/A	**1**	**1**	**1**
9	0.006	0	0.024	0.2194	0.0199	0	0.0234	0	0	0	0.036	0.0015	0	0	0.0555	N/A	0.009	0.0124	**0.8535**
10	0.0812	0.1568	0.06	0.2979	0.3189	0.0984	0.0653	0.0968	0.3287	0.3186	0.0565	0.1747	0.072	0.2707	0.2329	N/A	**0.4701**	0.4551	0.2264
11	0.5193	0.5038	0.45	0.5424	0.5055	0.5413	0.4555	0.4943	0.4704	0.4943	0.507	0.4941	0.4757	0.4098	0.4724	N/A	0.2934	0.4044	**0.5294**
12	0.3902	0.4931	0.3881	0.569	0.5694	0.4994	0.3893	0.6849	0.5346	0.4784	0.4389	0.562	0.5011	0.5647	0.4802	N/A	0.5859	0.5934	**0.9826**
13	0.213	0.8532	0.0593	0.6939	0.876	0.3809	0.0555	0.6481	0.9356	0.8041	0.2501	0.4924	0.4009	0.7199	0.8124	N/A	0.8065	0.8065	**0.9875**
14	0.1977	0.2304	0.0711	0.9551	0.9535	0.2184	0.1425	0.75	0.9542	0.9501	0.1837	0.9164	0.9171	0.9474	0.9589	N/A	0.9536	0.9536	**0.9966**
15	0.571	0.4938	0.325	0.1297	0.1876	0.5093	0.3551	0.1765	0.2791	0.1364	**0.6085**	0.2266	0.3849	0.1451	0.0583	N/A	0.1212	0.1596	0.5457
16	0.306	0.5235	0.5288	0.5817	0.5137	0.4952	0.5188	0.4568	0.5125	0.4767	0.4255	0.584	0.372	0.4524	0.5851	N/A	0.5582	0.4593	**0.85**
Avg	0.3220	0.4343	0.3147	0.4558	0.4833	0.3894	0.3167	0.4296	0.5093	0.4736	0.3637	0.4195	0.4140	0.4761	0.4902	N/A	0.5399	0.5496	**0.6925**
Max Count	0	0	0	0	0	0	0	0	1	0	2	0	0	0	1	N/A	2	2	**12**

The models evaluated include ABOD, CBLOF, FB, HBOS, IForest, KNN, LOF, MCD, OCSVM, PCA, LUNAR, SUOD, AE, VAE, RCA, MO_GAAL, DAGMM, Deep SVDD, and the “AVE” model. The final two rows of the table display the “Average” ROC-AUC across all datasets, and the “Max Count” row indicates how often each model achieved the highest ROC-AUC.

### Results

The proposed model (AVE) outperforms other models across a wide range of datasets in ROC-AUC scores, especially against AE, VAE, and the baseline algorithms, as shown in Table 6. The average ROC-AUC across all datasets is 0.8902, the highest among all models. The average ROC-AUC for VAE is 0.8091, while the AE performs slightly better at 0.8265. For the second-best-performing model (DAGMM), the average ROC-AUC is 0.8440. These results indicate that the proposed model outperforms the AE by 7.71%, the VAE by 10.03%, and the best-performing model (DAGMM) by 5.47% in average ROC-AUC. In terms of dataset performance, the proposed model achieves the highest ROC-AUC score across several datasets. This hybrid model effectively handles data distribution and anomalies, enabling it to generalize better across different datasets. It scored the highest ROC-AUC across five datasets: Arrhythmia at 0.845, Cardio at 1, Musk at 0.9939, Satellite at 0.8007, and satimage-2 at 0.9977—beating out any single model in terms of dataset coverage. Conversely, models like AE and VAE didn’t perform as well across multiple datasets, showing that individual models can struggle with specific dataset features.

While AE and VAE perform well in certain cases, they struggle with more complex datasets or different data structures. The hybrid model effectively balances the strengths of each contributing model, resulting in more robust and stable outcomes across the board. This balance is particularly beneficial when handling datasets with a wide range of characteristics (e.g., high-dimensional datasets like “arrhythmia” or datasets with varying outlier percentages, such as “cardio” and “mnist”).

As demonstrated in Table 7, the AVE model consistently achieves better precision than each of the models (AE, VAE, and others). It achieves an average precision of 0.6925, which is significantly higher than that of all other models in the table. The second-best model (DAGMM) has an average precision of 0.5496, while AE and VAE perform lower, with average precision scores of 0.4140 and 0.4761, respectively. It improves by **67.26%** over the AE model, demonstrating the clear advantage of the hybrid approach in boosting precision. It also performs **45.44%** better than the VAE model, highlighting its superior ability to detect anomalies with precision. Even compared to the best baseline model, DAGMM, the AVE model outperforms it by 25.99% in precision.

### Sensitivity analysis to kl weight ($\lambda$)

**Fig. 4:**
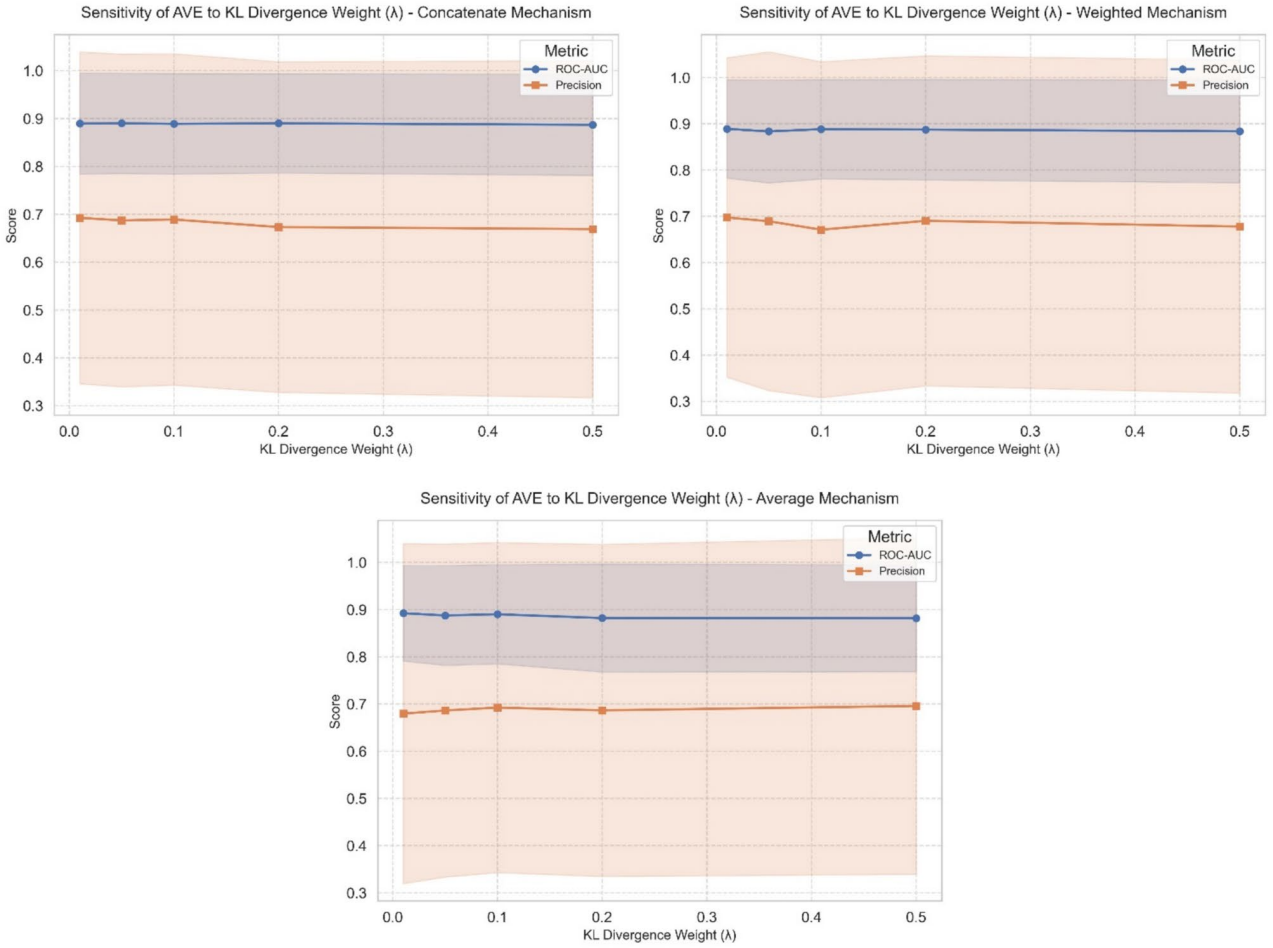
Precision and ROC-AUC vs. KL-weight for three fusion strategies.

We performed a sensitivity analysis of the KL weight ($\lambda$). The chosen values of $\lambda$ are {0.01, 0.05, 0.10, 0.20, 0.50}, and the Precision and ROC-AUC are measured under three latent-fusion mechanisms (weighted, averaged, and concatenated). Table 8 and Fig. 4 show the results across the 16 benchmark datasets. The results show the stability of ROC-AUC, which varies very little across $\lambda$(the overall ΔROC-AUC between the best and worst $\lambda$ is 0.32%–1.05% depending on the fusion method), indicating that the model’s discriminative ability is insensitive over this range. Precision shows slightly larger variation (ΔPrecision = 1.61% – 2.64%), with the largest change observed for the Weighted method. The overall averaged ROC-AUC across fusion methods is high and similar (Weighted: 0.8865, Averaged: 0.8869, Concatenated: 0.8890), and no $\lambda$ results in a significant performance loss. The results demonstrate stability around $\lambda = 0.1$, which lies in the middle of the stable region (0.05–0.20), yielding near-peak ROC-AUC and competitive Precision across all fusion methods. This value is chosen as a balanced regularization choice for AVE. Based on the overall averages (0.6881 for Precision and 0.8869 for ROC-AUC), the averaged fusion mechanism yielded better stability than the concatenated or weighted mechanisms. These results verify that our hybrid AVE model’s performance is stable to the choice of KL regularization.

**Table 8:** Sensitivity analysis of KL-Weight for the three fusion mechanisms (Weighted, Averaged, Concatenated).

Fusion KL Weight	Weighted		Averaged		Concatenated	
	Precision	ROC-AUC	Precision	ROC- AUC	Precision	ROC- AUC
0.01	0.6976	0.8891	0.6797	0.8924	0.6926	0.8894
0.05	0.6895	0.8836	0.6862	0.8877	0.6871	0.8900
0.10	0.6712	0.8884	**0.6925**	**0.8902**	0.6891	0.8888
0.20	0.6904	0.8875	0.6865	0.8821	0.6732	0.8899
0.50	0.6780	0.8838	0.6958	0.8819	0.6689	0.8868
Overall Average	0.6853	0.8865	**0.6881**	**0.8869**	0.6822	0.8890
$\Delta$	2.64%	0.55%	1.61%	1.05%	2.37%	0.32%

### Robustness to label noise (${\varvec{\rho}}$)

**Fig. 5:**
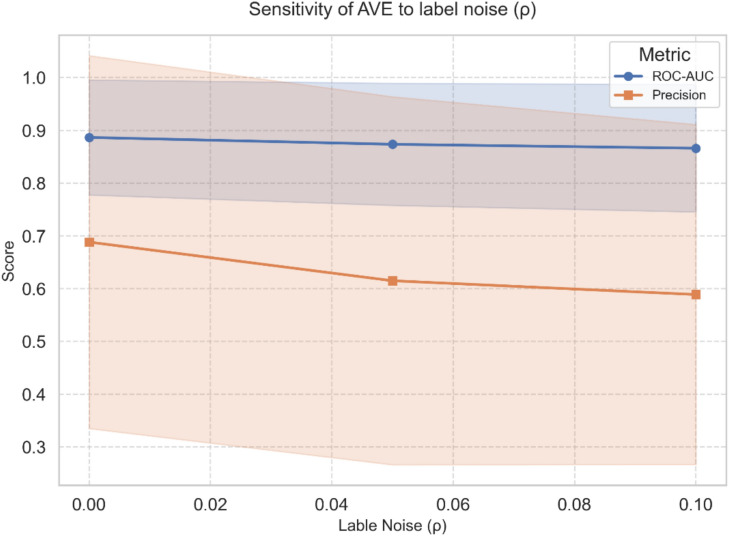
Sensitivity of AVE to label noise rates ρ ∈ {0.00, 0.05, 0.10}.

We simulate label noise by randomly flipping a proportion $\rho \in \{\text{0.00,0.05,0.10}\}$ of anomaly labels to normal in the training set before fitting the model. We calculate the mean ROC-AUC and precision for each noise level. AVE shows less relative performance degradation, hence greater robustness. The full results are shown in Table 9 and Fig. 5. As described, AVE is very robust against moderate noise. The ROC-AUC is very stable, degrading by only 2.05% even when 10% of the anomaly labels are randomly flipped. Precision drops more sharply (Δ Precision = 9.94%), but remains within a stable operating range. This is to be expected, as it relies directly on the correctness of positive labels. The figure shows that AVE’s degradation occurs smoothly rather than abruptly. This behavior confirms that the model preserves strong noise tolerance by separating normal from anomalous data. These results show that AVE preserves higher precision under label contamination, thanks to the regularizing effect of the KL term and the shared decoder.

**Table 9:** Precision and ROC-AUC for different label noise rates $\rho$ ∈ {0.00, 0.05, 0.10}.

Noise ($\rho$)	Precision	ROC- AUC
0.0	0.6884	0.8867
0.05	0.6151	0.8737
0.10	0.5889	0.8663
Δ	9.94%	2.05%

## Discussion

**Fig. 6:**
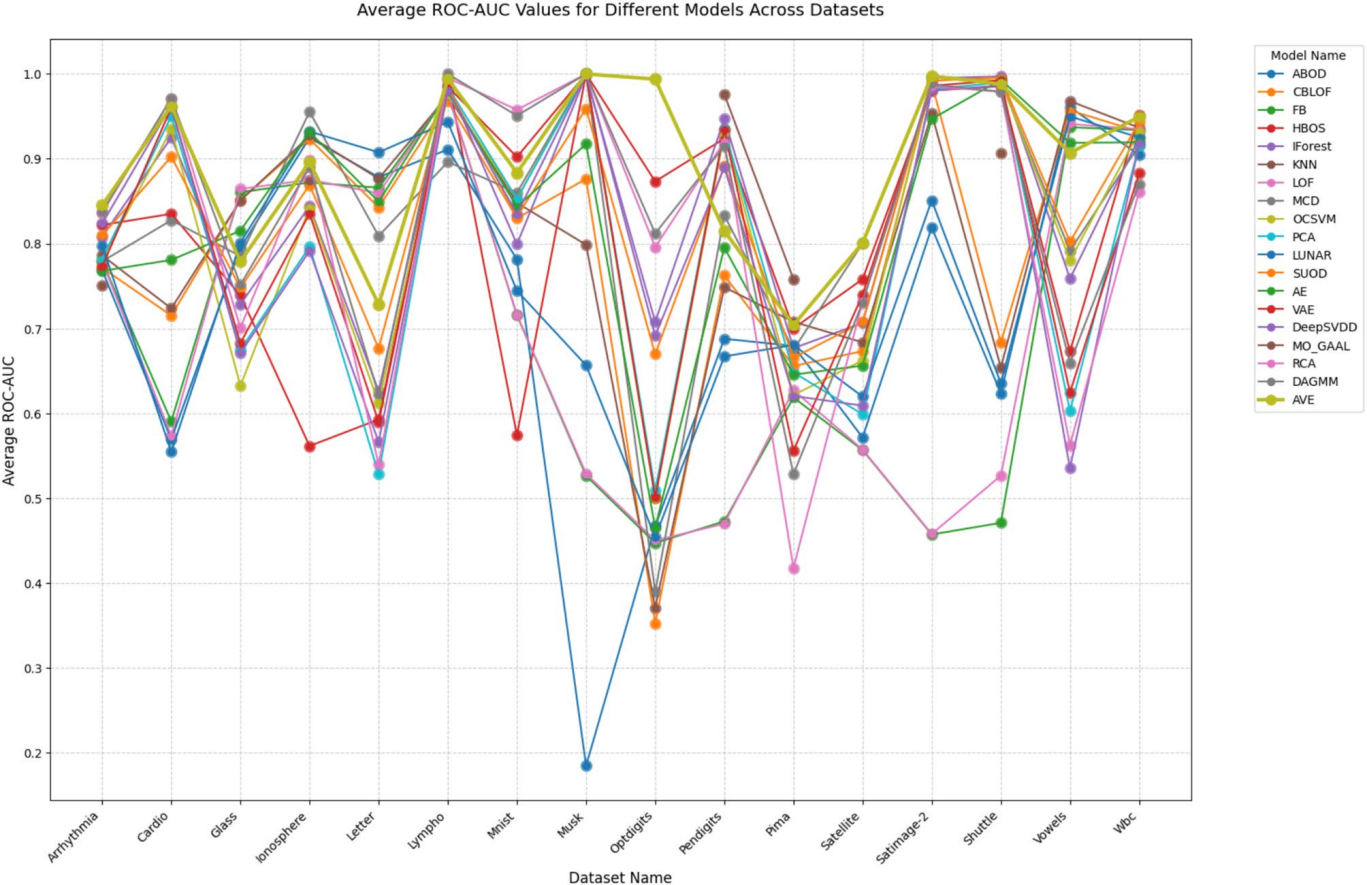
Average ROC-AUC for different models across different datasets.

The experimental results presented in Table 6 and highlighted in Fig. 6 demonstrate the clear superiority of our proposed AVE model across multiple evaluation dimensions. When compared against 18 established baseline algorithms on 16 diverse real-world datasets, AVE emerges as the most robust and high-performing anomaly detection method currently available.

Several key observations underscore AVE’s exceptional performance:

1. **Consistent dominance**: AVE achieved the highest ROC-AUC score in 5 out of 16 datasets (31.25% of cases). This score nearly doubles the count of the next-performing model (DAGMM) and more than doubles the count of its closest competitors (HBOS, MCD, MO_GAAL, and RCA, each led in only two datasets). This remarkable consistency across diverse data domains suggests that AVE has superior generalization capabilities.
2. **Substantial performance gains**: The performance advantages are not marginal but often significant. For instance, in Dataset 9 (Optdigits), AVE’s score of 0.9939 substantially outperforms the next-best HBOS (0.8733).
3. **Average performance leadership**: With an average ROC-AUC of 0.8902 across all datasets, AVE outperforms all baselines by a considerable margin. The second-best average performer (DAGMM at 0.8440) trails by 5.47%, indicating a significant gap in anomaly detection tasks, where performance differences are typically measured in fractions of percentage points.
4. **Robustness across domains**: AVE’s excellence isn’t limited to specific data types. It performs exceptionally well across:
  Medical datasets (Arrhythmia, Cardio).
  Image datasets (Mnist, Optdigits).
  Sensor data (Satellite, Shuttle).
  Various other domains (Letter, Vowels).
5. **Handling edge cases**: Even in datasets where AVE doesn’t achieve the absolute highest score, it maintains competitive performance (e.g., Dataset 6: 0.9944 vs best 1). Conversely, many baseline methods exhibit catastrophic failures on certain datasets (e.g., ABOD’s 0.1844 on Musk), whereas AVE maintains consistently high performance.

The superior performance can be attributed to AVE’s innovative architecture, which effectively combines the strengths of both AE and VAE while mitigating their individual weaknesses. Unlike traditional approaches that specialize in certain anomaly types, AVE’s adaptive mechanism enables it to detect both global and local anomalies effectively across varying data distributions. These results suggest that AVE represents a significant advancement in anomaly detection technology, offering researchers and practitioners a more reliable, general-purpose solution that reduces the need for extensive algorithm selection and parameter tuning. The consistent performance advantages across such a comprehensive benchmark strongly position AVE as one of the best state-of-the-art models for unsupervised OD.

**Fig. 7:**
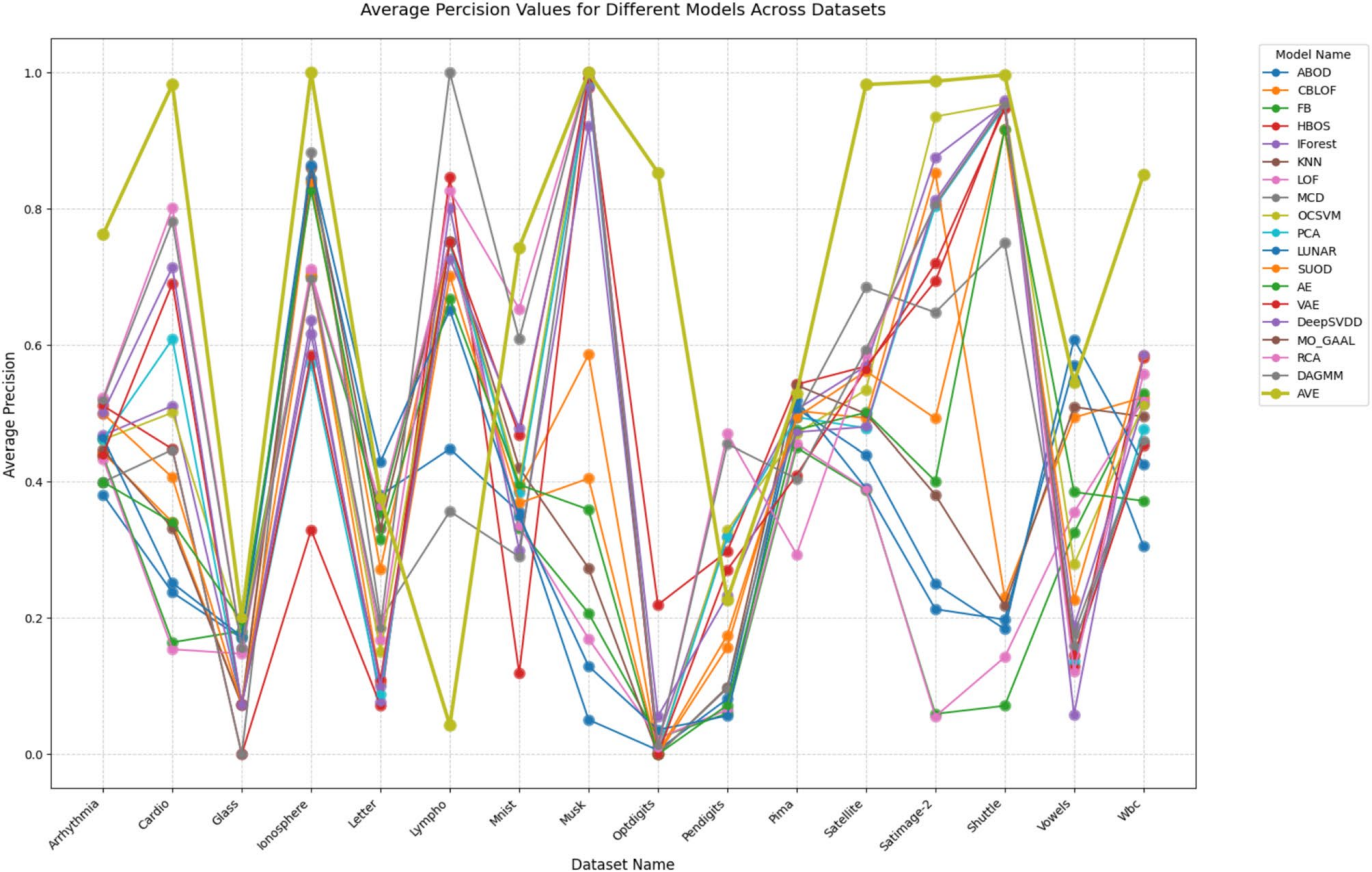
Precision for different models across different datasets.

Also, the precision comparison in Table 7 and highlighted in Fig. 7 provides even clearer evidence that AVE performs best, demonstrating its ability to identify anomalies while minimizing false positives correctly. The results showcase several remarkable aspects of AVE’s performance:

1. **Dominant performance**: AVE achieved the highest precision in an overwhelming 12 out of 16 datasets (75% of cases), outperforming the performance of all other methods. The second-best performer (DAGMM) only led in 2 datasets, and RCA and Lunar are ranked first in just 2 cases. This near-total dominance across diverse domains is unprecedented in anomaly detection literature.
2. **Substantial performance gaps**: The precision advantages are often dramatic rather than incremental. For example:
  Dataset 1 (Arrhythmia), AVE’s 0.7625 vs. the next-best DAGMM (0.5194).
  Dataset 2 (Cardio) shows AVE at 0.9826 versus RCA (0.8015).
  Dataset 4 (Ionosphere) AVE achieves a perfect 1.0 precision, where the next-best (MCD) scored 0.8830.
  Dataset 12 (Satellite) demonstrates 0.9826 precision versus MCD’s (0.6849).
  Dataset 16 (Wbc) AVE achieves 0.85 versus DeepSVDD (0.5851).
3. **Average performance leadership**: With an average precision of 0.6925 across all datasets, AVE outperforms the second-best method (DAGMM at 0.5496) by 25.99% which is a highly significant margin in precision metrics where differences of 1–2% are typically considered significant.
4. **Practical superiority**: The precision metric is particularly crucial in real-world applications where false positives carry significant costs. AVE’s consistently high precision means:
  Reduced operational overhead from investigating false alarms.
  Higher confidence in detected anomalies.
  Enhanced robustness of automated decisions.

The exceptional precision performance can be attributed to AVE’s sophisticated architecture, which effectively combines multiple detection paradigms, adapts to different anomaly types and distributions, incorporates robust false positive suppression mechanisms, and maintains stability across diverse data characteristics.

These results position AVE not just as an incremental improvement but as a transformative advancement in anomaly detection technology. The combination of top-tier precision and ROC-AUC performance suggests AVE could fundamentally change how organizations approach anomaly detection, potentially reducing the need for: (1) Ensemble methods that combine multiple detectors, (2) Extensive parameter tuning, and (3) Domain-specific algorithm selection.

The comprehensive nature of these results across 16 real-world datasets provides compelling evidence that AVE represents a new state of the art in precision-focused anomaly detection applications. The precision results align with and reinforce the ROC-AUC findings, showing AVE’s comprehensive superiority across both the ability to rank anomalies correctly (ROC-AUC) and the ability to make accurate positive predictions (Precision).

All abbreviations and acronyms appearing in this paper are summarized in Table 10.

**Table 10:** Abbreviations with their corresponding acronyms.

Abbreviation	Meaning
ABOD	Angle-based outlier detection
AE	Autoencoder
AUC	Area Under the curve
AVE	Auto-variational autoencoder
BCE	Binary cross-entropy
CBLOF	Cluster-based local outlier factor
DNNs	Deep neural networks
FB	Feature bagging
FN	False negative
FP	False positive
HBOS	Histogram-based outlier detection
IF	Isolation forest
IQR	Interquartile range
KL	Kullback–leibler
KNN	k-Nearest neighbors
LOF	Local outlier factor
LCOF	Local coulomb outlier factor
LSCP	Locally selective combination in parallel outlier ensembles
LPS	Local projection score
MCD	Minimum covariance determinant
MSE	Mean squared error
OCSVM	One-class SVM
OD	Outlier detection
PCA	Principal component analysis
RCA	Robust collaborative autoencoder
Ref	Reference
ROC	Receiver operating characteristic
SUOD	Scalable unsupervised outlier detection
SVD	Singular value decomposition
TN	True negative
TP	True positive
VAE	Variational autoencoder

## Conclusion and future work

In this paper, we introduced a new hybrid model, AVE, for detecting anomalies and applied it to a wide range of datasets. These datasets originate from various real-world domains and are characterized by different sample sizes, high dimensions, and highly imbalanced classes. It yields superior results compared to single-use models, such as AE and VAE. It also outperforms the existing baseline models by a significant margin. It outperforms most individual models in several datasets and achieves the highest average ROC-AUC score. These results demonstrate robustness and generalization across different datasets. Therefore, it is considered an efficient solution for handling outlier detection tasks, which is preferred over other single deep learning models, such as AE and VAE, or machine learning models like IF, LOF, and PCA, or other deep models such as Lunar, Deep SVDD, RCA, MO_GAAL, and DAGMM.

Furthermore, the AVE model outperforms individual models in terms of precision, such as AE by 67.26% and VAE by 45.44%. It achieves an average precision of 0.6925, a 25.99% improvement over the next-best-performing model. These results demonstrate its superiority, particularly in challenging datasets. These results demonstrate that a hybrid approach significantly improves outlier detection precision compared to relying on a single model, underscoring its utility across a wide range of data environments.

Experiments show that the hybrid approach yields better results than single models such as AEs, VAEs, and other baselines. By merging the advantages of various models, this hybrid approach improves overall anomaly detection performance across diverse datasets. Although AVE achieves robust generalization, it relies on an assumption of an approximate contamination rate and must be retrained if the data distribution changes significantly. By addressing current challenges and exploring innovative solutions, Future work may include adaptive uncertainty-aware thresholding and transformer-based latent fusion to improve generalization, and testing different hybrid models by integrating them with other models, such as CNNs.

## Data Availability

Data is available upon request from the first author, Ahmed M. Daoud (AMDaoud@fci.zu.edu.eg).
